# Causes and associated factors of mountain bike injuries in bike parks in western Austria: a two-season descriptive epidemiological study

**DOI:** 10.3934/publichealth.2026022

**Published:** 2026-04-01

**Authors:** Gerhard Ruedl, Alois Schranz, Patrick Salner, Vera Prünster, Martin Burtscher

**Affiliations:** 1 Department of Sport Science, University of Innsbruck, Innsbruck 6020, Austria; 2 medalp sportclinic, Imst 6460, Austria

**Keywords:** mountain biking, bike parks, epidemiology, injury, fractures, injury risk

## Abstract

Mountain bike terrain parks have become increasingly popular, particularly at ski resorts where chairlifts transport riders uphill to enjoy downhill cycling. These parks feature trails of varying difficulty with artificial obstacles designed for jumps and stunts, which contribute to a unique pattern of injuries. This retrospective epidemiological study analyzed mountain biking injuries in two bike parks in western Austria during the summer seasons of 2023 and 2024. The bike parks are located within two major ski resorts, where lifts transport riders and their bikes uphill. Injury data were systematically documented by bike patrols using an online reporting tool, while exposure data (number of biker visits and number of downhill rides) from one of the bike parks enabled the calculation of an injury rate. A total of 274 injured riders (81.8% male, mean age 30.2 ± 14.6 years), predominantly from Germany (65%), were recorded. Most injuries occurred between Friday and Sunday, primarily between 10 a.m. and 4 p.m., with over 90% occurring during downhill cycling and 15% during the first ride of the day. Falls due to rider errors accounted for 77.4% of injuries, while 20.8% were linked to jump landings. The shoulder/clavicle region was the most frequently injured site (33.7%), followed by the upper extremities (26%) and the head/face region (17%). Fractures were the most common diagnosis, accounting for 52% of cases. Approximately one-quarter of injured riders required helicopter evacuation. The calculated injury rate was 4 injuries per 1000 visits or 6 injuries per 10,000 rides. In conclusion, injuries in bike parks predominantly affect men and are more frequent on weekends. Falls during downhill cycling are the leading cause, with fractures and injuries to the shoulder/clavicle region being the most common. These findings highlight the need for targeted prevention strategies and optimized emergency services to improve safety in bike parks.

## 1. Introduction

Recreational sporting activities in the Austrian Alps have become increasingly popular among both locals and tourists throughout the year. While alpine skiing and snowboarding [1], ski touring [2], and tobogganing [3] are particularly popular during the winter months, mountain hiking [4] and mountain biking [5],[6] dominate the summer season. A representative survey conducted in Austria in 2020 among nearly 15,000 members of the Austrian Alpine Club revealed that 83% of respondents engage in mountain biking at least once a year, with 55% riding their mountain bikes more than 20 times a year [7]. However, the growing popularity of mountain biking has also resulted in a substantial increase in accidents, with the number of recorded incidents involving mountain bikes tripling between 2006 and 2018 in Austria [6].

In recent years, mountain bike terrain parks have emerged as a growing trend, particularly in ski resorts, where chairlifts are repurposed to transport riders uphill, enabling them to descend the slopes on their bikes [8]. This allows riders to maximize the amount of downhill riding they can achieve in a single day while minimizing the physical effort required for uphill climbs [9]. These terrain parks are designed with man-made obstacles and features that encourage riders to perform jumps and stunts, which increases the risk of injury [8]. For example, a study by Aitken et al. [10] reported that approximately 45% of injuries occurred in the freeride area of a mountain bike facility.

Injuries and fatalities sustained during mountain sports activities, regardless of the season, are generally attributed to a single inciting event, such as a fall or a collision [11]. However, these events often result from a complex interplay of intrinsic risk factors, such as age, sex, and skill level, and extrinsic risk factors, such as the type of equipment used and environmental conditions, such as weather and surface quality [11]. According to Bahr and Krosshaug's four-step framework for injury prevention research [11], understanding the specific circumstances of accidents is critical for developing evidence-based preventive measures to enhance the safety of mountain bikers in this increasingly popular sport. Additionally, data such as the day and time of the accident, and the type and severity of the injuries, are crucial for planning and organizing rescue services, including in bike parks during the summer months [12].

To date, numerous studies have investigated the occurrence of injuries, their potential causes, and contextual factors in the Austrian Alps, focusing on activities such as alpine skiing and snowboarding, ski touring, tobogganing, and mountain hiking [1]–[4]. However, research is lacking on the potential causes and associated factors of recreational mountain biking accidents in Austrian bike parks, with further investigation needed to address this gap in knowledge. Therefore, this retrospective study aimed to investigate potential causes and associated factors of recreational mountain biking accidents resulting in injury in two major bike parks in western Austria.

## 2. Materials and methods

### 2.1. Study design and participants

This study was designed as retrospective analysis of documented mountain biking injuries in two major bike parks in the federal state of Tyrol, Austria, during the summer seasons of 2023 and 2024. The bike parks are located within two major ski resorts and have lifts to transport riders and their bikes uphill. Additionally, like ski patrols in winter, the ski resorts deployed bike patrols in summer to administer first aid in the event of an injury.

The study was conducted in accordance with the Declaration of Helsinki and was approved by the University of Innsbruck's Institutional Board for Ethical Questions in Science (Certificate No. 86/2025). Informed consent was waived because bike parks are mandated to routinely document accidents resulting in injuries.

For every event necessitating on-site medical care, medically trained bike patrollers documented participants' sex, age, nationality (Austria, Germany, Switzerland, or other), helmet use (yes/no), and the day and time of day (before 10 a.m., 10–12 p.m., 12–2 p.m., 2–4 p.m., 4–6 p.m., or after 6 p.m.) of the injured bikers using an online reporting tool. The following were also recorded: accident site (downhill trail, bike park, forest road, other), steepness of the trail (flat, medium, steep), pre-injury activity (cycling uphill, cycling downhill, cycling on flat terrain, unknown, other), cause of injury (fall caused by riding error, fall during landing after jump, collision with other biker, heart attack, other), and transportation mode of assessing medical care (injured person independently, mountain rescue/rescue vehicle, helicopter, other).

Following the classification by Koehne et al. [13], the primary anatomical body regions affected by the most severe injuries were categorized as the head/face, shoulder/clavicle, upper extremity, lower extremity, and trunk. Regarding the diagnosis of the most severe injury, a distinction was made between contusion, sprain, crushing injury, abrasion, open wound, dislocation, fracture, ligament and muscle injury, concussion, other brain injury, and other types of injury. In cases of multiple injuries, the most severe diagnosis was determined using the following hierarchy: fracture > dislocation > concussion or other brain injury > ligament and muscle injury, etc.

Additionally, a bike park provided not only the number of registered injuries but also exposure data, i.e., the number of first entries (“biker visits”) into the bike park during the two seasons, as well as the number of rides determined using the electronic ski lift system. Based on this data, an injury rate per 1000 visits or per 10,000 downhill rides can be calculated.

### 2.2. Statistics

Descriptive statistics are presented as means ± standard deviation, as absolute and relative frequencies, as well as injury incidence per 1000 visits and 10,000 downhill rides with Poisson 95% confidence interval (CI), and were analyzed using SPSS 27.0 (IBM Corporation, Armonk, NY, USA).

## 3. Results

A total of 274 injured mountain bikers (81.8% male) with a mean age of 30.2 ± 14.6 (range: 2–68) years were recorded by the bike patrollers of the two bike parks over two seasons from July 2023 to October 2024. The majority of injured bikers were from Germany (64.5%), followed by Austria (12.8%), Switzerland (8%), and other countries (14.7%). Overall, 268 (98%) injured mountain bike riders were wearing helmets.

**Figure 1.**
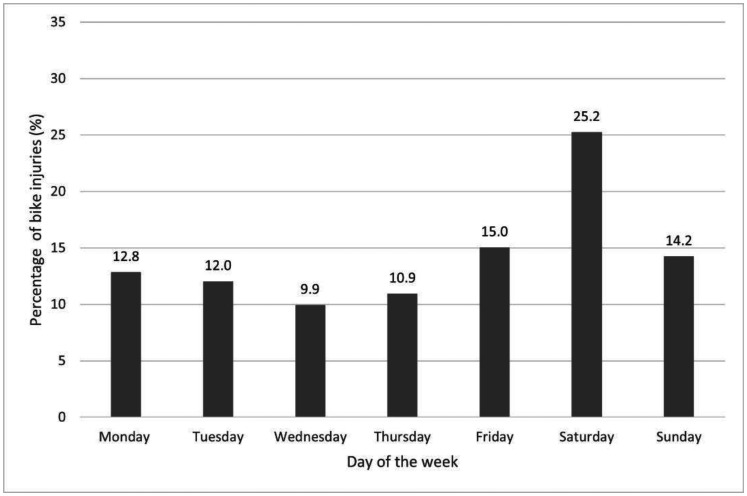
Percentage of mountain bike injuries by day of the week.

**Figure 2.**
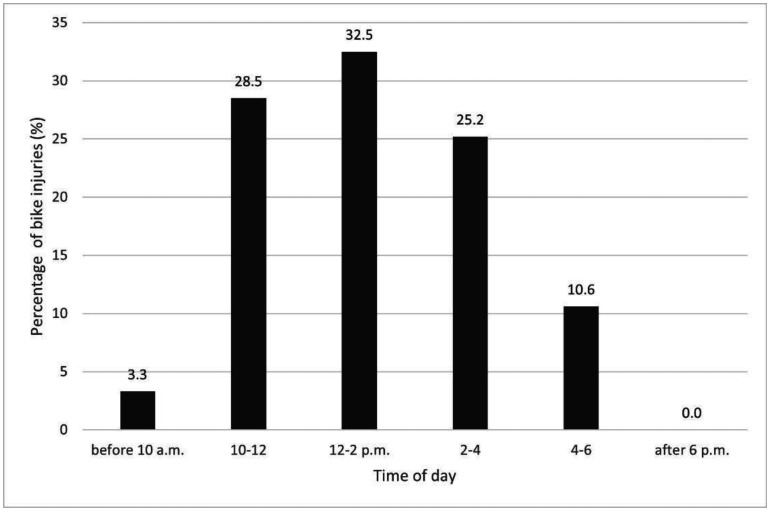
Percentage of mountain bike injuries by time of day.

Figure 1 shows the percentage of injuries by day of the week, and Figure 2 shows the distribution of injuries based on the time of day. More than 50% of injuries occurred between Friday and Sunday, with the majority (86.2%) happening between 10 a.m. and 4 p.m.

A total of 37 out of 250 patients (14.8%) reported that the injury occurred during their first ride of the day (data were missing for 24 patients).

Table 1 presents the injury frequencies categorized by accident location, slope steepness, pre-injury activity, cause of injury, and transportation mode of assessing medical care.

**Table 1.** Absolute (N) and relative (%) frequencies of accident locations, slope steepness, pre-injury activity and cause of injury, and transportation mode of assessing medical care among 274 injured mountain bikers.

	N (%)
Accident location
Downhill trail	73 (26.6)
Bike park	194 (70.8)
Forest road	3 (1.1)
Others	4 (1.5)
Steepness of the slope
Flat	98 (35.8)
Medium	146 (53.3)
Steep	30 (10.9)
Pre-injury activity
While cycling uphill	3 (1.1)
While cycling downhill	256 (93.4)
While cycling on flat terrain	9 (3.3)
Unknown	3 (1.1)
Other	3 (1.1)
Cause of injury
Fall caused by a riding error	212 (77.4)
Fall during landing after a jump	57 (20.8)
Collision with another biker	3 (1.1)
Heart attack (internal emergency)	1 (0.4)
Other	1 (0.4)
Transportation mode of assessing medical care
Independently by the injured person	18 (6.6)
Mountain rescue/rescue vehicle	188 (68.6)
Helicopter	67 (24.5)
Other	1 (0.4)

Table 2 presents the frequencies of multiple sustained injuries, as well as the injured anatomical body regions and the diagnosis of injuries. Approximately one-fifth of the mountain bikers suffered from multiple injuries. The shoulder/clavicle region was the most frequently affected anatomical area, representing one-third of all injuries. The most common injury diagnoses were fractures, accounting for 51.6%, followed by concussions at 12.8%.

The calculated average injury rate over the two seasons was 4.1 injuries per 1000 biker visits (95% CI: 3.6, 4.6) and 6.3 injuries per 10,000 downhill rides (95% CI: 5.5, 7.1).

## 4. Discussion

In line with a recent study by Woyke et al. [6], which examined mountain biking injuries in the Austrian Alps between 2006 and 2018, women accounted for less than a fifth of injuries. Similarly, other mountain biking studies have shown that males account for over 80% of injuries in some cases [13]–[17].

**Table 2.** Absolute (N) and relative (%) frequencies of multiple injuries, anatomical body parts, and injury diagnoses among 274 injured mountain bikers.

	N (%)
Multiple injuries, yes	56 (20.6)
Missing value	2
Injured body region
Head/face	46 (16.8)
Shoulder/clavicle	92 (33.7)
Upper extremity	71 (26.0)
Lower extremity	22 (8.1)
Trunk	42 (15.4)
Missing value	1
Diagnosis of injury
Contusion	29 (10.6)
Sprain	5 (1.8)
Crushing injury	3 (1.1)
Abrasion	3 (1.1)
Open wound	21 (7.7)
Dislocation	22 (8.1)
Fracture	141 (51.6)
Ligament and muscle injury	6 (2.2)
Concussion	35 (12.8)
Other brain injury	2 (0.7)
Other	6 (2.2)
Missing value	1

From an economic perspective, winter and summer tourism is important in the Austrian federal state of Tyrol, with its ski resorts hosting two of the largest bike parks in the country. It is therefore not surprising that most mountain biking injuries involve tourists from other countries (e.g., from Germany or Switzerland), predominantly occurring at weekends, with 39.4% of injuries recorded on Saturdays and Sundays. Consistent with these findings, Ashwell et al. [9] reported that 39.5% of all bike park injuries occurred on Saturdays and Sundays.

In this study, one-fourth of injured individuals were evacuated by helicopter. Helicopter evacuation is typically employed in cases of severe injury or where terrestrial rescue is not feasible due to terrain conditions. By way of comparison, the average frequency of helicopter transport for mountain biking injuries in the Austrian Alps between 2006 and 2018 was 18% [6]. This discrepancy may be attributed to the present study's focus on mountain bike parks, which include a significant number of downhill trails. Downhill biking is generally associated with a higher risk of severe injury compared to other disciplines [18].

To the best of our knowledge, this is the first study to examine whether injuries occurred during the first ride of the day. Around 15% of injured riders sustained their injuries during this time. By contrast, Becker et al. [19] reported that most downhill injuries (58%) occurred midway through the day, with the remainder evenly distributed between the start (21%) and end (20%) of the day. This finding is of particular interest as it could encourage bike parks to implement targeted preventive measures such as recommending or offering structured warm-up routines, bike safety checks, or skill improvement courses.

More than 90% of injuries occurred during cycling downhill. Bonte et al. [15] reported that most mountain bike accidents occurred on moderate (46%) or steep (30%) descents. One 60-year-old male mountain biker suffered a heart attack while cycling uphill. The average age of individuals sustaining traumatic mountain biking injuries in this study is approximately 30 years. Evidence suggests that advanced age and male sex are associated with sudden cardiac arrest during recreational activities in mountainous regions [20]. For example, most fatal mountain biking incidents in Austria have been found to be non-traumatic, typically being attributed to cardiovascular disease, which predominantly affects males with a median age of 56 years [5].

The most common cause of injury was falling because of a riding error, accounting for more than three-quarters of cases. Becker et al. [19] consistently reported that 72% of downhill mountain biking injuries were attributed to riding errors. Losing control of the front wheel is often associated with MTB accidents resulting in an injury [16]. In addition, Kronisch and Pfeiffer [21] stated that errors in judgment and riding technique, such as riding at excessive speeds, being inattentive, riding beyond one's skill level, misjudging the situation, performing incorrect braking maneuvers, and being intoxicated, can lead to injuries. In the present study, only three injuries were reported as resulting from a collision with another mountain biker. Similarly, Ashwell et al. [9] observed that collisions between riders in a mountain bike park rarely resulted in clinical care being sought.

Approximately one-fifth of the injured mountain bikers in this study sustained more than one injury. By contrast, more than half of the participants in the study by Ashwell et al. on mountain bike park injuries presented with multiple injury diagnoses [9]. These differing results may potentially be attributed to variations in timing (2023 vs. 2009), location (Austria vs. Canada), and data collection method (bike rescue services vs. hospitals) in the context of mountain biking injuries. Regarding the injury pattern, similar findings were reported by a recent study by Teramoto et al. [22], identifying the shoulder/clavicle region as the most common injury location, representing 32.7% of all injury events. Fractures are the most common injury diagnosis in mountain bikers, which is consistent with the existing literature [16],[17],[22]–[24]. Furthermore, fractures are reported to be the leading cause of hospitalization due to mountain biking compared to other types of injury [12],[23],[24].

A total of 80.8% of fractures affected the shoulder/clavicle region (48.2%) and the upper extremity (32.6%) (data not displayed). By comparison, Ashwell et al. [9] reported that 74.2% of documented fractures among mountain bikers injured in a bike park affected the upper extremities, including the shoulder and clavicle. Furthermore, Breulmann et al. [14] recently investigated mountain bike downhill injuries in an Austrian region across two seasons, in 2020 and 2021. They reported a total of 259 fractures to the upper extremities, 42.1% of which were clavicular fractures [14].

The causes of shoulder girdle injuries and injuries to the upper extremity may be related to the type of fall: typically, a forward fall over the handlebars of the mountain bike with an outstretched arm to break the impact or a lateral fall where the arm is used for protection [9]. Recently, a video analysis of more than 500 mountain bike injuries identified the forward over-the-bars with 55% as the most common crash scenario, followed by the sideways ejection with 12% [15]. In the predominant “over-the-bars” scenario, riders are ejected over the handlebars because of sudden deceleration, which frequently occurs on moderate descents (53.2%) or after poorly executed jump landings (64.4%) [15]. Tumbles represented the most frequent type of ground impact (64.7%), with shoulder girdle injuries (39.7%) and upper limb injuries (35.6%) being the most prevalent [15].

To calculate the injury rate during the study period, one bike park provided exposure data (the number of entries and rides), which was collected using the ski lift's electronic tracking system. The resulting injury rate was determined to be 4 injuries per 1000 visits and 6 injuries per 10,000 downhill rides. By way of comparison, an earlier study by Aitken et al. [10], conducted at a Scottish mountain bike center, reported an injury rate of 1.54 per 1000 visits. These differences may be attributed not only to the varying regions and time periods but also to the fact that the bike parks examined in this study are in alpine terrain with trails starting above 2000 m above sea level. What does this injury rate mean for mountain bike tourists visiting these bike parks in summer? An injury rate of four injuries per 1000 visits corresponds to one injury per 250 visits. Assuming a mountain bike tourist spends ten days in the bike park during the summer months, the statistical injury rate would be one injury per 25 mountain biking sessions (250/10). However, it should be noted that the actual injury rate often depends on multiple intrinsic (e.g., gender, skill level, fitness, and risk-taking behavior) and extrinsic (e.g., equipment and environmental conditions) risk factors [11].

Several limitations must be considered when interpreting this study on mountain biking injuries in bike parks. For example, selection bias is a significant issue, as the study population may only include individuals who sought medical assistance within the park. Riders with minor injuries who do not report them may be underrepresented, which could lead to certain cases being underestimated. Data quality and consistency may be limited because bike-patrol reporters did not receive standardized training, the online reporting tool lacked validation checks, and no inter-rater reliability assessment or audit of random cases was performed. Injury diagnoses were determined by medically trained bike patrollers; however, these assessments were not verified against medical records. Furthermore, the results may not be comparable or generalizable to other bike parks due to differences in trail design, difficulty levels, and safety measures between the two parks studied. In addition, because exposure data were available only for one park, the estimated injury rate is not generalizable to both parks. Reporting bias may also occur, as data collection relies on the voluntary participation of bike park rescuers, meaning a complete record of all reported injuries cannot be guaranteed. Confounding variables, such as the rider's skill level, risk-taking behavior, and the use or non-use of protective equipment, may also influence the likelihood of injury. The study may also be subject to temporal limitations, as data collected during a specific season or over a short period may not capture long-term trends or seasonal variations in injury patterns. Conversely, to the best of our knowledge, there are only a few older studies on injuries in mountain bike parks, and none are specifically from bike parks in the Austrian Alps.

## 5. Conclusions

In conclusion, the frequency and patterns of injuries occurring in bike parks, as demonstrated, will support the implementation of targeted preventive measures and contribute to optimizing emergency services.

## Use of AI tools declaration

The authors declare they have not used Artificial Intelligence (AI) tools in the creation of this article.
